# Prodromal Parkinson's disease: hype or hope for disease-modification trials?

**DOI:** 10.1186/s40035-022-00286-1

**Published:** 2022-02-21

**Authors:** Philipp Mahlknecht, Kathrin Marini, Mario Werkmann, Werner Poewe, Klaus Seppi

**Affiliations:** Department of Neurology, Innsbruck Medical University, 6020 Innsbruck, Austria

**Keywords:** Neuroprotection, Preclinical, Prevention, Epidemiology, Probability, Randomized controlled trial, Preventive, Neuroprotective

## Abstract

The ultimate goal in Parkinson's disease (PD) research remains the identification of treatments that are capable of slowing or even halting the progression of the disease. The failure of numerous past disease-modification trials in PD has been attributed to a variety of factors related not only to choosing wrong interventions, but also to using inadequate trial designs and target populations. In patients with clinically established PD, neuronal pathology may already have advanced too far to be modified by any intervention. Based on such reasoning, individuals in yet prediagnostic or prodromal disease stages, may provide a window of opportunity to test disease-modifying strategies. There is now sufficient evidence from prospective studies to define diagnostic criteria for prodromal PD and several approaches have been studied in observational cohorts. These include the use of PD-risk algorithms derived from multiple established risk factors for disease as well as follow-up of cohorts with single defined prodromal markers like hyposmia, rapid eye movement sleep behavior disorders, or PD gene carriers. In this review, we discuss recruitment strategies for disease-modification trials in various prodromal PD cohorts, as well as potential trial designs, required trial durations, and estimated sample sizes. We offer a concluding outlook on how the goal of implementing disease-modification trials in prodromal cohorts might be achieved in the future.

## Background

Parkinson’s disease (PD) continues to be clinically defined by a set of cardinal motor features anchored on the presence of bradykinesia and at least one additional motor symptom out of rest tremor or rigidity [1, 2]. Neuropathological evidence suggests that these motor symptoms only emerge after 40%–60% of neurons in the substantia nigra have been lost and striatal dopamine levels are reduced by 60%–70% [3, 4]. Even prior to the onset of nigral neurodegeneration, extranigral Lewy-body pathology is believed to affect the peripheral autonomic nervous system, the caudal brainstem, as well as the olfactory bulb [5] and may correlate to a variety of early nonmotor symptoms that have been shown to antedate the first appearance of classical motor signs [2, 6–8]. Along these lines, population-based or other cohort studies have established a significantly increased risk of developing PD in otherwise healthy subjects with hyposmia, constipation, depression, excessive daytime somnolence, and idiopathic rapid eye movement (REM) sleep behavior disorder (RBD) [9–15]. For people with idiopathic RBD, conversion into a clinically overt neurodegenerative disorder, most commonly PD or dementia with Lewy-bodies (DLB), has been estimated to occur at a rate of around 6% per year [16], strongly suggesting that a large proportion of those subjects are actually in a stage of prodromal Lewy-body disease. This and other evidence leave little doubt that the onset of PD pathology in the nervous system occurs much earlier than is captured by the current definition of clinical disease onset (Table 1) [17, 18].

**Table 1:** Conceptual stages of Parkinson’s disease (PD)

Phases of PD [17, 18]	Clinical status	Pathology	Comments
Phase 1—preclinical PD	No clinical signs or symptoms	PD-specific pathology assumed to be present	Supported by biomarkers (genetic, molecular, and/or imaging)
Phase 2—prodromal PD	Early nonmotor symptoms ± early subtle motor symptoms	Extranigral PD pathology (Braak stages 1 and 2) ± nigral PD pathology (< 40%–60% cell loss; Braak Stage 3)	Research criteria defined based on clinical nonmotor markers (± motor markers) and nonclinical biomarkers. There may be various levels of certainty [18]; probable prodromal PD defined at ≥ 80% probability (sufficiently certain for disease-modification trials) [24] and possible prodromal PD (lower, but still substantial likelihood, e.g., 30%–80%)
Phase 3—clinically established PD	Classical motor manifestations are present	Nigral PD pathology (> 40%–60% cell loss; Braak stages 3 to 6)	Current clinical diagnostic criteria based on motor syndrome are met [1]; ± a variety on nonmotor symptoms may be present due to extranigral extension of PD pathology

The ultimate goal of identifying subjects in the prodromal stages of PD is to offer therapies that are able to modify the course of the disease in that they delay or even prevent the development of clinically established PD and related disability (Panel 1). Indeed, the failure of trials of disease-modifying interventions in PD to date may in part be due to the fact that pathology in established PD is too advanced for the respective treatments to be effective [19, 20]. If this is the case, then targeting prodromal PD would offer much greater chances of success for future trials. Also, disease-modification trials targeting subjects in the prodromal phase of the disease would not have to account for the confounding effects of established symptomatic PD therapies, which has been a significant issue in past trials in early PD.

In this review, we discuss potential target populations and recruitment strategies for disease-modification trials in prodromal PD as well as potential trial designs, required trial durations, and estimated sample sizes. We offer a concluding outlook on how the goal of disease-modification trials in prodromal cohorts might be achieved in the future.

**Table:** 

Panel 1: Conceptual framework of disease-modification in Parkinson’s disease (PD)
*- Disease-modification*
Any therapy that alters the clinical course (‘natural history’) of a disease can be regarded as ‘*disease-modifying’*. Such a broad definition would also include symptomatic therapies for PD as they reduce the severity and functional impact of motor and non-motor symptoms and thus positively influence the progression of disability. However, in regulatory science (and in the context of this review), the term disease-modification is used in a narrower sense, i.e., for a therapy that is capable of positively influencing the course of the disease by biological mechanisms that revert disease-specific pathophysiological changes [20, 21]. Such mechanisms may include not only slowing or halting the otherwise progressive loss of monoaminergic neurons (i.e., neuroprotection), but also improving downstream signaling processes and compensatory responses. *- Neuroprotection* The term *‘neuroprotection’* was introduced to capture beneficial (protective) effects of an intervention on neuronal survival and function. While such neuroprotective effects can be expected to translate into clinically detectable disease-modification, the presumed underlying biological effect on neuronal survival cannot be proven during lifetime without validated biomarkers that are closely linked to the disease-specific neuronal pathology. In the context of PD, such biomarkers are currently lacking. Previous trials have used molecular imaging markers of pre-synaptic nigro-striatal terminal function as a surrogate for measuring nigral cell loss, but this technique is not able to distinguish between functional effects of an intervention and effects on neuronal survival. *- Disease-prevention* The term ‘*disease-prevention’* is tempting to use in the context of trials in prodromal cohorts, as prevention of clinically established PD can be seen as the ultimate goal of disease-modifying interventions in such subjects. However, there are conceptual problems even here, given that prodromal disease stages are, by definition, already a disease state. Nonetheless, in a broader sense, the term can be used to describe effects of an intervention that forestall the development of clinically overt PD. In this review, we include such effects under the umbrella term of (early) *disease-modification.* *- Regulatory definitions of disease-modification in PD* The European Medical Agency requires a two-step procedure to demonstrate disease-modification in PD – first, a delay in clinical measures of disease progression should be shown and, second, an effect on the underlying pathophysiological process which correlates to a meaningful, and persistent changes in clinical function [22]. The Food and Drug Administration (FDA) has not published guidance related to PD. However, in their latest guidance related to drug development in early Alzheimer’s disease, the term ‘disease-modifying’ has been replaced by ‘persistent effect on disease course’ that should be accompanied by a ‘direct effect on the underlying disease pathophysiology’ [23].

## Identifying prodromal PD as a target population for disease-modification trials

Based on a solid body of evidence from prospective studies of risk factors for the development of PD [9–15], two principal approaches to identifying subjects with high likelihood of harboring prodromal PD have been used. They include multifactorial screening algorithms for PD risk on the one hand and approaches targeting single specific risk or prodromal markers like hyposmia or RBD with subsequent enrichment steps on the other hand. Carrier status for monogenic PD genes is a third and much narrower starting point.

To date, there are some published risk scores that have been developed in relation to their predictive performance for incident PD [24–27]. The two best studied and most comprehensive screening multi-item algorithms are the online-based PREDICT-PD risk score [27, 28] and the Movement Disorders Society (MDS) research criteria for prodromal PD [24, 29]. The PREDICT-PD algorithm incorporates remotely-assessable early non-motor features and risk factors combined into a risk score that in the original study was associated with incident PD during follow-up over 3 years with a hazard ratio of 4.4 [27]. Two validation attempts showed weaker, but significant associations of the score with incident PD, with odds ratios of 1.3–2.1 [30, 31]. An enhanced PREDICT-PD risk score has recently been developed that integrates three additional markers and showed better accuracy in predicting PD compared with the original score [32].

**Fig. 1:**
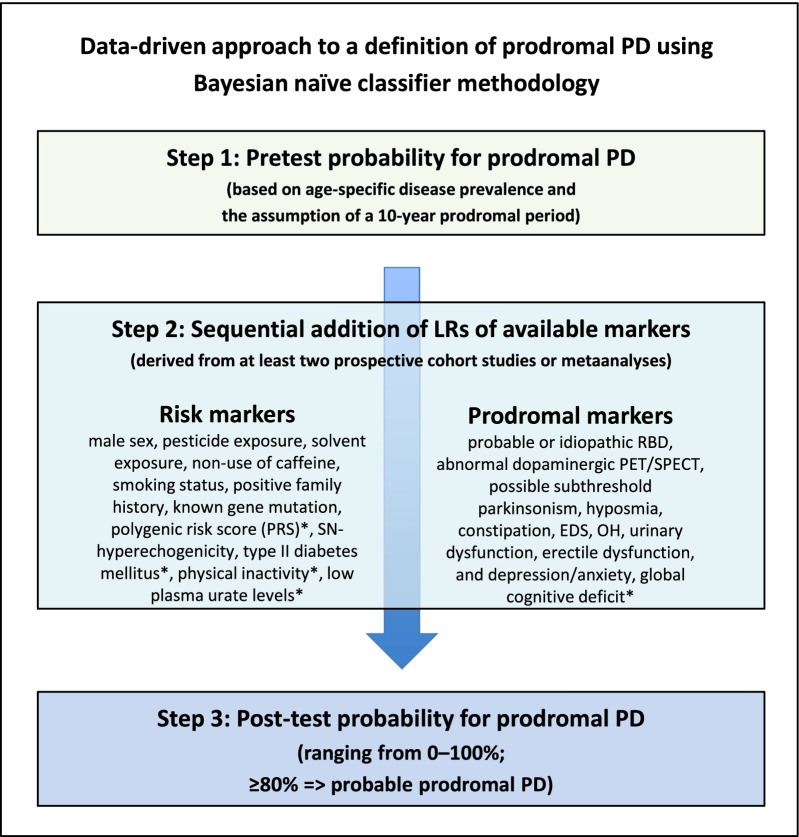
Schematic structure of MDS research criteria for prodromal PD. The approach by the MDS task force entails 1. assessment of pretest probability for prodromal PD based on age, 2. the sequential addition of LRs of various risk and prodromal markers, and 3. the calculation of post-test probabilities using the above information (see Berg  et al. 2015 [24] for further details). Modified from Mahlknecht et al. 2018 [34], with kind permission from Wiley. * Markers added with the 2019 update of the MDS research criteria for prodromal PD [29]. EDS, excessive daytime somnolence; LR, likelihood ratio; OH, orhtostatic hypotension; PD, Parkinson’s disease; SN, substantia nigra

The MDS criteria were specifically designed to calculate probabilities of prodromal PD in a given subject, where an 80% threshold is the cut-off for a diagnosis of ‘probable prodromal PD’ (see Fig. 1 for schematic structure of the MDS criteria) [24]. They are novel in that they do not only make use of single risk factors or specific combination of a selection thereof. In fact, they aim at including all established PD risk and prodromal markers, thereby generating a comprehensive picture of an individual’s risk profile that is integrated in a probability score (similar to risk scores in cardiovascular medicine). In 2019, a first update of the criteria was presented, which incorporated new evidence for risk and prodromal markers already included and supplemented them with four new markers [29]. While prospective evaluations of the criteria and their update are awaited, evidence from retrospective applications of the criteria to existing longitudinal population-based cohorts seems promising [33–36]. They consistently reported high specificity and negative predictive values (NPV) in identifying incident cases of PD, while sensitivity and positive predictive values (PPV) varied substantially, dependent on the type of study population (enriched risk *vs* population-based), depth of marker assessment, and length of follow-up time. Interestingly, a high predictive accuracy of the MDS criteria has also been detected in a cohort study in 121 patients with idiopathic RBD (PPV 81%; for conversion into PD or dementia with Lewy bodies over 4 years) [37] and a cohort of leucine-rich repeat kinase 2 (*LRRK2*) mutation carriers (PPV 47%–67%) [38].

The second type of approach uses single established PD risk factors for an initial screen with subsequent enrichment for high risk of ‘conversion’ into clinically PD. Such strategies make use of sequential screening steps of increasing specificity, starting with easily assessable and ideally sensitive markers – candidate markers may include positive family history, hyposmia, subtle motor impairment, probable RBD, or combinations thereof [39–42]. Subsequent enrichment steps would use additional clinical biomarkers such as neuroimaging, polysomnography or genetic assessment. The prospective PRIPS (Prospective evaluation of Risk factors for Idiopathic Parkinson’s Syndrome) study has found that by using prescreening for age (> 50 years), primary screening for positive family history and/or hyposmia, and secondary screening for substantia nigra hyperechogenicity, one out of 16 positively screened participants developed PD over 3 years (PPV of 6.1%) compared to one out of 135 in the original cohort [41]. This is clearly insufficient to meet the requirements of a feasible disease-modification trial with incident PD as an endpoint. Similarly, the prospective Parkinson associated risk syndrome (PARS) study used a first screening step of olfactory function assessment with University of Pennsylvania Smell Identification Test (UPSIT) mailings in subjects aged > 50 years and found that of 152 hyposmic individuals followed-up over 4 years, 19 (12.5%) developed PD [43]. However, when adding dopamine transporter (DAT) single-photon emission computed tomography (SPECT) as an enrichment screen, 14 of 21 with a DAT-SPECT deficit (and hyposmia) developed PD, resulting in a PPV of 67%.

Patients with idiopathic RBD seem to represent a particularly useful target population as the majority of patients with idiopathic RBD (> 80%) will convert into a neurodegenerative disorder – most commonly PD, PD dementia (PDD), or DLB [11]. Annualized conversion rates to PD (52%) or DLB (44%) have averaged ~ 6% per year in the most recent multicenter RBD longitudinal series [16]. However, median latency to conversion can be as long as 12 to 14 years [11, 12]. This seriously limits the feasibility of trials with a phenoconversion endpoint in idiopathic RBD patients. Several studies have found that the addition of other clinical markers such as hyposmia [13, 16, 44], abnormal color vision [16, 44], or subtle motor dysfunction [13, 16, 44] can result in high conversion rates over shorter periods of time. This would, however, reduce the size of available cohorts. Potential solutions for this problem would be multicenter efforts to collect large-enough patient samples and/or to enlarge existing patient cohorts by population-based RBD screening (such as in the recently started *Luxembourg's National Sleep Survey*; https://www.rbd.lu, last accessed 12/2021). Another caveat for idiopathic RBD patients in this context is that conversion not only occurs to PD but with similar frequency also to DLB and more rarely to multiple system atrophy (MSA), such that trials in idiopathic RBD patients would inform on disease-modification in Lewy Body disorders and synucleinopthies in general.

**Fig. 2:**
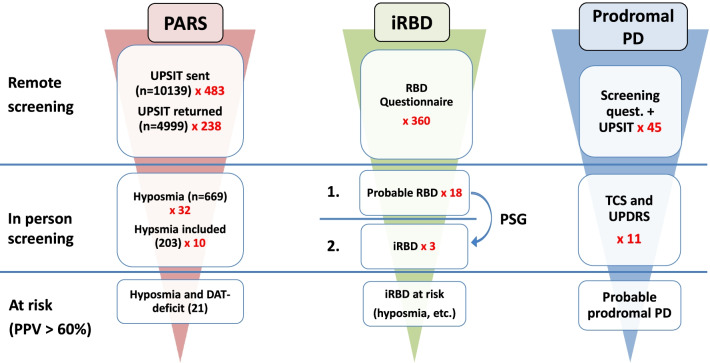
Potential screening strategies for target populations for disease-modification trials in the general community assuming three different target populations (PPV > 60%). First column: individuals with hyposmia and DAT-Deficit; real numbers from the PARS study that used olfactory testing as first remote screening step and DAT-Scan as a second screen are presented [43]. These include losses to follow up. Second column: patients with idiopathic RBD and further marker(s) that indicate increased risk for early conversion as hyposmia, abnormal color vision, or subtle motor dysfunction (approximately one third of idiopathic RBD patients) [13, 44]; the identification of idiopathic RBD patients from the general community is modeled using a questionnaire as a first remote screening step (assuming a prevalence of probable RBD of 5%) [46, 48] and polysomnography as a second screening step (assuming that approximately one sixth of probable RBD cases are confirmed as having idiopathic RBD) [45, 46]. Third column: Individuals with probable prodromal PD according to the MDS research criteria as modeled from data of the prospective population-based Bruneck Study [34]; remote screening includes a questionnaire-based assessment and brief odor-identification test. The model envisages that all participants reaching a post-test probability for prodromal PD of > 10% (one quarter of participants) are invited for the in person screening including a motor examination and transcranial sonography. Estimated numbers necessary for remote screening are derived from the prevalence of probable prodromal PD (i.e. 2.2%). Please note that only multipliers in the first column account for losses to follow-up, whereas the ones in the second and third columns do not

Figure 2 illustrates potential screening strategies in the general community for target populations for disease-modification trials assuming three different approaches, in which substantial conversion rates to clinically overt PD (> 60% over 5 or less years) have been described. The numbers of individuals from the general elderly population needed to be screened to obtain such samples are significant. Such numbers would perhaps be feasible to screen with online approaches similar to the one used in the PREDICT PD algorithm [27], with published strategies for RBD screening in the population [45–47], or risk scores using data originating from primary care presentations and general practitioners [26].

Finally, asymptomatic carriers of mutations in monogenic PD genes, e.g. *LRRK2,* or in risk genes like glucocerebrosidase (*GBA*), identified as first-degree relatives of PD patients, also represent potential prodromal target populations for disease-modification trials [19, 49, 50]. Such cohorts are attractive, since they have a defined underlying molecular disease mechanism that can be targeted with specific (‘personalized’) interventions. However, since penetrance is incomplete, the application of additional biomarkers that can indicate conversion into manifest PD in shorter time-frames would be necessary for this group as well. The MDS criteria could again be a suitable instrument for this purpose. When applied to a multicenter cohort of 120 *LRRK2* mutation carriers (first-degree relatives of *LRRK2* PD patients), of whom only 10 converted to PD over 5 years, the cut-off for prodromal PD status yielded a PPV of 47%–67% [38]. Despite these obstacles, an obvious hope is that eventual disease-modifying mechanisms identified in such genetic groups are generalizable and that developed treatments might subsequently be successfully applied to sporadic PD patients.

### Role of biomarkers in identifying prodromal PD

Even if comprehensive instruments such as the MDS prodromal criteria enabled detection of individuals with prodromal PD at high accuracy, it would be desirable to have a highly specific confirming biomarker as a last step before inclusion of participants into disease-modification trials in order to reduce false-positives to a minimum. Ideally, such a biomarker would also indicate disease progression and therefore represent an additional outcome measure as a surrogate marker of disease-modification. Previous studies have mainly used molecular imaging of the nigrostriatal dopaminergic system, in particular DAT imaging. In the PARS study, hyposmic individuals with a baseline DAT deficit (below the 65% age-expected lowest putamen binding ratio) experienced a futher 4-year 20% decline in DAT binding (equaling to an annual 5% reduction similar to early PD) compared with 4%–5% in those with only intermediate or no DAT-deficit [43]. Future imaging candidates may include promising, but yet to be validated magnetic resonance imaging (MRI)-based biomarkers that are specific for PD (e.g., nigrosome-1, free-water, or neuromelanin imaging of the substantia nigra) [51–56] and show change over time. Real-time quaking-induced conversion (RT-QuIC) identifying pathologically folded α-synuclein at high sensitivity constitutes another emerging and potentially suitable biomarker for this purpose. In one study, RT-QuIC-identified pathogenic α-synuclein in the cerebrospinal fluid (CSF) had a sensitivity of 95% and a specificity of 98% in discriminating PD, DLB, and idiopathic RBD from controls, atypical parkinsonian disorders and Alzheimer’s disease [57]. The same method was also found to be predictive of development of PD or DLB in idiopathic RBD patients [58]. Alternative approaches for biospecimen collection for RT-QuIC analysis are skin biopsies with a similarly high diagnostic yield [59–61] and, although less sensitive, olfactory mucosa by nasal swabs [62].

## Trial design issues for disease-modification trials in prodromal PD

Traditionally, disease-modifying trials have been performed in early disease stages of established PD and have, so far, mostly shown negative or inconclusive results despite numerous attempts applying different treatments, trial designs, and outcome variables [2, 19, 20]. The most prominent examples in the form of high-quality and rigorous randomized controlled trials (RCTs) assessed disease-modifying properties of selegiline and tocopherol (DATATOP) [63], levodopa (ELLDOPA and LEAP) [64, 65], pramipexole (PROUD) [66], rasagiline (ADAGIO) [67], creatine (NET-PD) [68], and exenatide (EXENATIDE-PD) [69]. Trial designs comprised of simple two-arm double-blind approaches with time to need for levodopa [63], or change in motor status as assessed with the UPDRS after 2-week [64] or 12-week [69] washout period as endpoints, or delayed-start approaches with change in motor status according to the UPDRS as endpoint [65–67]. Some of these trials additionally assessed biological imaging outcomes as surrogates for disease-modification, such as change in presynaptic nigrostriatal dopaminergic dysfunction with radiotracer imaging [64, 66]. Multiple factors may have contributed to the many failures in this field, including the type of intervention, target population, clinical trial design, choice of the primary outcome measures, and trial duration.

When planning disease-modification trials in prodromal PD populations, some additional conceptual issues and caveats have to be taken into account. For the most intuitive endpoint of conversion into PD, a simple long-term RCT trial with time-to-event analysis would be suitable. However, definition of phenoconversion has some challenges and there are reasons for choosing continuous outcomes such as motor or non-motor progression (see following sections), for which both classical parallel-group or delayed-start designs could be used [20, 70]. However, using progression of motor or non-motor features also poses some issues since—unlike in early PD—progression rates of disease-related clinical dysfunction in motor or non-motor domains are poorly defined and there is hardly any sensitive and validated non-clinical progression biomarker. These obstacles result in many uncertainties regarding suitable trial endpoints and durations.

## Endpoints

Table 2 delineates examples for potential outcomes of disease-modification trials in prodromal PD. Conversion to clinically established PD seems to represent the most obvious primary endpoint. It has already been used in many observational studies of prodromal PD cohorts [13, 33, 34, 37, 38, 44], some of which also calculated sample sizes required for disease-modifying trials (see next section) [13, 16, 34, 44]. The assessment of the exact disease onset in such high-risk populations, however, is rather subjective and observer-dependent and comes with an inherent risk of poor standardization and heterogeneity in multi-center trials [71].

**Table 2:** Examples of potential endpoints for disease-modification trials in prodromal PD

Type of marker	Outcome (endpoint)	Availability of data from studies in prodromal cohorts	Availability of data from studies in early PD patients	Advantages	Disadvantages
Clinical marker	Phenoconversion to clinically defined PD	Assessed in many unselected and preselected population-based cohorts [27, 33, 34, 38, 43], assessed in iRBD [13, 37, 44, 73]	NA	Represents the essence of prophylactic therapies	May require long trial duration of 5 or more years, difficulties in determining the exact time point of phenoconversion
	Motor progression (e.g., motor parts of the MDS-UPDRS/UPDRS)	Assessed in population-based [74] and iRBD cohorts [75, 76]	Assessed in early PD patients [77–79]; MDS-UPDRS Part II scores increased 1.0 point per year, and Part III scores increased 2.4 points per year, steepest increment observed in year 1	Shorter intervals due to continuous outcome	Motor progression might be slow in the early “premotor” phase (but accelerates shortly before diagnosis and in very early PD)
	Non-motor progression	Assessed in population-based [74] and iRBD cohorts [75, 76]	Assessed in early PD patients [78, 80]	Non-motor progression may be faster in “premotor” stages than motor progression	Heterogeneous outcome
	Progression of the prodromal PD score	Assessed in TREND [33], PMPP [81], and in a *LRRK2* cohort [38]; results suggest the progression may only be seen in true prodromal PD cases that may indeed go on to develop PD	NA	Universal outcome that seems to be very different in true prodromal cases versus healthy controls	Large interindividual variability [33]
Imaging marker	Decline in striatial dopaminergic binding	Assessed in the PARS cohort (5% decline per year) [43] and in iRBD patients (6% decline per year) [82]	Assessed in early PD patients in the PPMI cohort [79] and in disease-modification trials [64, 66]	Objective, quantifiable, correlates with disease severity	Represents surrogate marker, expensive, requires multiple resources
	Progression of MRI markers [53]	NA	Free-water imaging [83] Neuromelanin imaging [84, 85]	Objective, quantifiable, correlates with disease severity	Represents surrogate marker, expensive, requires specialized MRI
Biochemical marker	Change in alpha-synuclein in CSF	Assessed in individuals with prodromal PD in the PPMI cohort [86]	Assessed in the PPMI cohort [86, 87]	Lower in PD patients and individuals with prodromal PD	No change over time observed in early PD patients; no correlation with disease progression or ongoing neurodegeneration in MDS-UPDRS and DAT-Scan results; contradicting results
	RT-QuIC-assessed alpha-synuclein	Assessed only as disease state biomarker [58, 62]; but not yet as progression biomarker	Assessed only as disease state biomarker [57, 59–61]; but not yet as progression biomarker	Highly sensitive	Not yet assessed in term of progression marker. Specialized lab equipment

CSF, cerebrospinal fluid; DAT, dopamine transporter; iRBD, idiopathic REM-sleep behaviour disorder; LRRK2, leucine-rich repeat kinase 2; MDS, Movement Disorders Society; MRI, magnetic resonance imaging; NA, not assessed; PMPP, Progression Markers in the Premotor Phase study; PPMI, Parkinson’s progression marker initiative; TREND, Tuebinger evaluation of Risk factors for Early detection of NeuroDegeneration study; UPDRS, Unified Parkinson’s Disease Rating Scale; RT-QuIC, Real-time quaking-induced conversion

Clinical rating scales providing continuous indices of progression of motor and non-motor dysfunction have been standard outcome measures of past disease-modification trials in early PD, but almost none have revealed significant treatment effects over periods of 1–2 years. While this may have been a problem of the interventions, the problem of using these outcomes will be even greater in prodromal PD where there is very limited information on their rates of progression. Compared to disease-modification trials in early PD, there is an even greater need for sensitive and meaningful progression biomarkers in trials in the prodromal stage of the disease. DAT imaging is an example, for which there are published progression rates from the PARS study (i.e., ~ 5% decline per year in putaminal binding). Other measures may include advanced MRI-based analyses like neuromelanin or free-water imaging of the substantia nigra. The most attractive imaging biomarker would require the availability of alpha-synuclein position emission tomography (PET) tracers [72]—similar to beta-amyloid imaging in Alzheimer’s disease. Alpha-synuclein seeding assays in CSF or tissues like skin or olfactory mucosa have shown very promising diagnostic performance in PD and also prodromal cohorts with idiopathic RBD [57, 58, 62], but so far they have not been tested or validated regarding their sensitivity to measure disease progression. Efforts are underway to make alpha-synuclein aggregation seeding assays quantifiable for use as a marker for biological drug effects, which would be of great significance for early-stage clinical trials (for example for alpha-synuclein immunization trials).

In general, large prospective natural-history studies in the general population and prodromal PD cohorts will more clearly delineate progression of clinical and biomarker indices over time. To this end, the prospective Parkinson Progression Marker’s Initiative (PPMI) has been expanded to include prodromal PD cohorts including subjects with hyposmia or RBD who meet a threshold for DAT deficit as well as genetic mutations in *LRRK2*, *GBA*, or *SNCA* (https://www.ppmi-info.org/study-design; last accessed 12/2021)***.*** It is hoped that prodromal PPMI and other prospective studies will provide robust natural history data of progression of clinical and biomarker indices in prodromal PD.

## Sample sizes and trial duration

The basic key considerations that influence decisions on sample size and duration of a PD trial are summarized in Panel 2. With regard to disease-modification trials in neurodegenerative disorders such as PD, there are particular challenges that are further amplified when targeting prodromal disease stages. PD is generally slowly progressive, requiring longer trial durations in the range of at least 2–3 years in order to detect significant changes in clinically relevant outcomes. This would likely have to be even longer to detect effects on progression in prodromal PD. Prodromal PD populations with high likelihood of phenoconversion like subjects with idiopathic RBD or asymptomatic carriers of *LRRK2* mutations also have a low prevalence compared to clinically established early PD.

Nonetheless, there are viable strategies that could potentially be used for recruitment. Figure 2 shows three examples for target population recruitments from the general community that have a > 60% likelihood of reaching the endpoint of conversion into clinically identifiable PD or DLB. For an outcome of phenoconversion, the number of events can be increased either by choosing higher-risk patients, by increasing the follow-up time, or by increasing the sample size. Most observational studies in prodromal PD have been performed in an attempt to identify rates of conversion into manifest PD (± DLB) with a given set of risk/prodromal markers in various populations. Some of these studies have also reported estimations for sample sizes for disease-modification trials that would use a phenoconversion endpoint: in our own population-based study, sample size estimates at 80% power in an intention-to-treat approach ranged from 108 to 540 subjects with probable prodromal PD depending on trial duration (3–5 years) and effect size of the agent (30%–50%) [34]. For example, in an intention-to-treat approach, a total of 540 and of 294 individuals with probable prodromal PD would be required to have an 80% chance to detect a 30% decrease in the primary outcome measure of conversion to PD for 3 years and 5 years, respectively. The numbers for a more efficient intervention at 50% effect size would be substantially lower (190 and 108 individuals, respectively). However, our estimations rely on a low number of converters only, may therefore not be precise, and must be reassessed in future prospective population-based studies. There are other studies that assessed sample sizes in idiopathic RBD cohorts. Idiopathic RBD is thought to be the most specific marker with the highest PPV with a conversion rate of 80% into a Lewy-body disorder. However, the median latency to conversion into clinically defined PD or DLB can be as long as 12–14 years [11, 12], seriously limiting the feasibility of disease-modification trials in idiopathic RBD cohorts. In fact, in the largest idiopathic RBD cohort published so far, Postuma et al*.* found conversion rates of 6% per year with 52% converting into PD and 44% into DLB [16]. Various markers were assessed for their potential to identify those patients at high risk of early conversion and, thus, those suitable for inclusion into disease-modification trials. Based on the time-to-event analysis, the authors estimated that 366 unselected idiopathic RBD patients per arm would be needed for a 2-year trial at 80% power to find a 50% reduction in disease phenoconversion [16]. The most powerful single selection procedure was abnormal motor testing, which reduced sample size to 166–197 patients; however, only one third of idiopathic RBD patients had abnormal testing, thus excluding two thirds of patients from such a trial. Abnormal olfaction was present in two thirds of patients and reduced sample size to 247–262, and a probable prodromal PD status according to MDS criteria was present in three quarters of patients and yielded sample sizes of 282–301 per group.

In another recent monocenter analysis on progression of prodromal markers in patients with idiopathic RBD who also met the definition of probable prodromal PD status as per MDS criteria, motor progression on the UPDRS was the marker associated with lowest sample size requirements, but was not more powerful than time to phenoconversion with both endpoints requiring approximately 240 patients per arm (2-year trial at 80% power and 50% efficacy) [76]. The most efficient design was a time-to-event analysis using milestones of motor and cognitive decline (e.g., 126 per group when using rise in UPDRS III ≥ 4 points or a MoCA decline ≥ 3 points) as primary endpoints.

**Table:** 

Panel 2: Trial duration and sample size—basic key considerations
Sample sizes for clinical trials should be large enough and trial durations long enough to avoid the type II or β error (i.e., falsely accepting the null hypothesis H0; meaning that a significant difference could be detected using larger samples or longer trial durations). Sample-size estimations depend on 5 factors: 1. The type I or α error (i.e., the likelihood of false positive results by falsely rejecting the null hypothesis H0), which is usually set at 5%. 2. The power equaling to 1-β, which is usually set at 80% or 90%. 3. The estimated effect size of the intervention (e.g., 30% reduction of worsening or occurrence of an outcome measure). 4. The assumed dropout rate: the intention-to-treat estimations do also consider dropouts in studies and are therefore more conservative as compared with per protocol analyses. This is particularly important for disease-modification trials in PD, which require long durations. 5. The variability in the occurrence of the outcome—for disease-modification trials with a binary endpoint (e.g., time to phenoconversion) this is equivalent to the estimated percentage of trial participants that potentially meet the endpoint. This obstacle can be overcome by evidence from robust natural history data in large prodromal cohorts.

## Conclusions and outlook

Although PD stands out among the neurodegenerative diseases by the availability of highly efficacious symptomatic therapies, none of these treatments can prevent the inevitable long-term progression of disease into serious motor and non-motor disability [2, 88, 89]. After more than 20 years of frustration with failed ‘neuroprotection’ or ‘disease-modification’ trials, there is now new hope that a breakthrough may eventually become possible. This optimism is mainly driven by major advances that have occurred in two research areas over the last decade and have opened new opportunities to finally achieve successful disease-modification in PD. One is related to the ability to identify subjects in the prodromal or ‘pre-diagnostic’ stage of PD, where underlying molecular pathologies may still be reversible or at least more responsive to targeted interventions. As outlined in this article, there are still important challenges to overcome when planning trials in such populations, but an important beginning has been made. The other highly significant progress made in recent years concerns a growing understanding of the molecular pathways involved in PD pathogenesis—primarily driven by unravelling the genetic architecture of PD. This has revealed a multitude of novel targets for disease-modification, and a growing number of interventions addressing such targets have been and are being tested in clinical trials in early PD [19, 20]. But again, there have also been disappointments with some of these new targeted interventions like alpha-synuclein antibodies or substrate reduction therapies for glucocerebrosidase enzyme in *GBA*-positive patients with PD [90–92], suggesting a need for alternative approaches. Among these, targeting prodromal instead of early clinically diagnosed PD seems to offer enhanced potential for success. However, a number of obstacles have still to be removed as the field gets ready to embark on this route.

One of this is the painful lack of progression biomarkers that are sensitive to detect intervention effects within shorter time frames than are currently needed for clinical outcomes and that are closely linked to clinical progression and outcome. The availability of alpha-synuclein PET tracers could potentially respond to that need [72], similar to beta-amyloid imaging in Alzheimer’s disease. The discussion around this has gained momentum after the recent FDA approval for the anti-amyloid Alzheimer drug adecanumab that was primarily based on intervention effects on the imaging biomarker. However, the resulting controversy about this regulatory decision has also highlighted the issues around the use of biomarker outcomes that are not underpinned by concurrent effects on clinical measures [93]. Even if a one-year trial in prodromal PD could demonstrate efficacy of an intervention on a validated progression biomarker, there will still be a need for a longer controlled extension to demonstrate translation into clinically relevant effects.

A second major challenge to address relates to concerns that the ‘one drug fits all’ approach that has been used in all disease-modification trials in PD so far, ignores patient heterogeneity in terms of underlying molecular pathologies and may thus obscure the disease subtype-specific efficacy of an intervention [94]. Future trials in prodromal PD will have to deal with this, for example, by selecting target populations with specific molecular pathologies for corresponding target-specific interventions—like the use of kinase-inhibitors in *LRRK2* mutation carriers [50]. In order to roll out such ‘personalized’ strategies beyond prodromal monogenic PD subtypes, there is still a largely unmet need for a biomarker-supported platform to identify pathogenetic disease subtypes. In the context of sporadic PD, potential subtypes of PD have been defined in the PPMI cohort of early PD patients differentiating between a ‘diffuse malignant’ type, a ‘mild motor predominant’, and an ‘intermediate’ type [95]. The malignant PD phenotype is characterized by higher motor deficits and non-motor symptom burden in terms of cognitive impairment, RBD, and dysautonomia, and is associated with faster progression and higher risk for major disease milestones and death [95, 96]. Similarly, a ‘body-first’ PD subtype with early dysautonomia and RBD and a ‘brain-first’ subtype of PD have recently been described according to the presumed site of initiation of PD pathology [97–99]. While being of high interest to the field of prodromal PD [94], these concepts have so far only been assessed in a few studies in manifest PD and idiopathic RBD and their relevance for the planning of future disease-modification trials is still unclear.

At the other end of the spectrum, successful disease-modification in prodromal PD might eventually require drug combinations addressing multiple targets, not unlike what is common practice in primary prevention of cardiovascular disease. The latter example also highlights the potential role of life-style modifications in reducing disease risk, an area that has been explored to some extent in neurodegenerative diseases like PD or Alzheimer’s disease, where physical and mental activity, dietary, drinking and smoking habits, as well as control of vascular co-morbidities have been shown to modify disease risk [100–102]. While there are significant challenges regarding controlled prospective studies of the effects of such factors on the evolution of disease in prodromal subjects, they would nonetheless be essential for individual patients [103] and for evidence-based PD risk-management at the public health level.

Integrating screening for PD risk and prodromal PD into public health programs is the ultimate goal once effective therapies to delay or prevent progression to manifest PD will have become available. However, many of the required screening criteria in the public health context [104, 105] are not yet fully met for PD. Importantly, there are several ethical issues that need consideration also in relation to research efforts of identifying prodromal PD subjects. ‘Medicalization’ is a social phenomenon when individuals, who receive a screening invitation, may become worried on the possibility of having the disease [106, 107]. Overdiagnosis is another well-known problem in the oncology literature for controversial cancer screening tests which aim at early cancer detection such as mammography and prostate-specific antigen screening [107], and refers to the detection of subclinical disease (sometimes called pseudodisease) which would not have become manifest clinically in someone’s remaining lifetime [108, 109]. Depending on a given health care system, a positive screening result, i.e. diagnosis of prodromal PD, can have significant impact on the access to health care or life insurances [110]. Moreover, diagnostic accuracy of a screening procedure is of major concern, and predictive values for manifest PD of current diagnostic criteria for prodromal PD are still suboptimal. False-positive results of a screening procedure give rise to unnecessary further diagnostic tests and create worries of having a disease that may never manifest. Examples are not only subjects with false positive prodromal risk score results, but also patients with idiopathic RBD that are now commonly medically defined as harboring PD, PDD/DLB, or MSA, although a minority may remain free of any symptoms of such disease for decades or their entire life-span. Risk disclosure strategies should therefore take factors such as prodromal status (e.g., RBD-positive *versus* RBD-negative) into account [111]. Inviting subjects to join trials or other research in prodromal PD requires careful individualized counseling and an option for psychosocial support.

Despite all the challenges and unmet needs discussed in this review, the PD field has probably never been as close to a fulfillment of James Parkinson’s prophecy of 1817 as it is in 2022: “*there appears to be sufficient reason for hoping that some remedial process may ere long be discovered, by which, at least, the progress of the disease may be stopped*”.

## Data Availability

Not applicable.

## Abbreviations

CSF: Cerebrospinal fluid
DAT: Dopamine transporter
DLB: Dementia with Lewy-bodies
FDA: Food and Drug Administration
*GBA*: Glucocerebrosidase gene
*LRRK2*: Leucine-rich repeat kinase 2 gene
MRI: Magnetic resonance imaging
MSA: Multiple system atrophy
NPV: Negative predictive values
PD: Parkinson’s disease
PDD: Parkinson’s disease dementia
PARS study: Parkinson associated risk syndrome study
PET: Position emission tomography
PPV: Positive predictive values
PPMI: Parkinson Progression Marker’s Initiative
RT-QuIC: Real-time quaking-induced conversion
RBD: Rapid eye movement sleep behavior disorder
UPDRS: Unified Parkinson’s disease rating scale
UPSIT: University of Pennsylvania Smell Identification Test

## References

[1] Postuma RB, Berg D, Stern M, Poewe W, Olanow CW, Oertel W (2015). MDS clinical diagnostic criteria for Parkinson’s disease. Mov Disord.

[2] Poewe W, Seppi K, Tanner CM, Halliday GM, Brundin P, Volkmann J (2017). Parkinson disease. Nat Rev Dis Prim.

[3] Fearnley JM, Lees AJ (1991). Ageing and Parkinson’s disease: substantia nigra regional selectivity. Brain.

[4] Greffard S, Verny M, Bonnet AM, Beinis JY, Gallinari C, Meaume S (2006). Motor score of the unified Parkinson disease rating scale as a good predictor of Lewy body-associated neuronal loss in the substantia nigra. Arch Neurol.

[5] Del Tredici K, Braak H (2012). Lewy pathology and neurodegeneration in premotor Parkinson’s disease. Mov Disord.

[6] Schrag A, Horsfall L, Walters K, Noyce A, Petersen I (2015). Prediagnostic presentations of Parkinson’s disease in primary care: a case-control study. Lancet Neurol.

[7] Mahlknecht P, Seppi K, Poewe W (2015). The concept of prodromal Parkinson’s disease. J Parkinsons Dis.

[8] Pont-Sunyer C, Hotter A, Gaig C, Seppi K, Compta Y, Katzenschlager R (2015). The onset of nonmotor symptoms in Parkinson’s disease (The ONSET PDStudy). Mov Disord.

[9] Ross GW, Petrovitch H, Abbott RD, Tanner CM, Popper J, Masaki K (2008). Association of olfactory dysfunction with risk for future Parkinson’s disease. Ann Neurol.

[10] Chen H, Shrestha S, Huang X, Jain S, Guo X, Tranah GJ (2017). Olfaction and incident Parkinson disease in US white and black older adults. Neurology.

[11] Iranzo A, Tolosa E, Gelpi E, Molinuevo JL, Valldeoriola F, Serradell M (2013). Neurodegenerative disease status and post-mortem pathology in idiopathic rapid-eye-movement sleep behaviour disorder: an observational cohort study. Lancet Neurol.

[12] Schenck CH, Boeve BF, Mahowald MW (2013). Delayed emergence of a parkinsonian disorder or dementia in 81% of older men initially diagnosed with idiopathic rapid eye movement sleep behavior disorder: a 16-year update on a previously reported series. Sleep Med.

[13] Mahlknecht P, Iranzo A, Högl B, Frauscher B, Müller C, Santamaría J (2015). Olfactory dysfunction predicts early transition to a Lewy body disease in idiopathic RBD. Neurology.

[14] Fereshtehnejad SM, Yao C, Pelletier A, Montplaisir JY, Gagnon JF, Postuma RB (2019). Evolution of prodromal Parkinson’s disease and dementia with Lewy bodies: a prospective study. Brain.

[15] Mahlknecht P, Stockner H, Marini K, Gasperi A, Djamshidian A, Willeit P (2020). Midbrain hyperechogenicity, hyposmia, mild parkinsonian signs and risk for incident Parkinson’s disease over 10 years: a prospective population-based study. Parkinsonism Relat Disord.

[16] Postuma RB, Iranzo A, Hu M, Högl B, Boeve BF, Manni R (2019). Risk and predictors of dementia and parkinsonism in idiopathic REM sleep behaviour disorder: a multicentre study. Brain.

[17] Stern MB, Lang A, Poewe W (2012). Toward a redefinition of Parkinson’s disease. Mov Disord.

[18] Berg D, Postuma RB, Bloem B, Chan P, Dubois B, Gasser T (2014). Time to redefine PD? Introductory statement of the MDS Task Force on the definition of Parkinson’s disease. Mov Disord.

[19] Poewe W, Seppi K, Marini K, Mahlknecht P (2020). New hopes for disease modification in Parkinson’s Disease. Neuropharmacology.

[20] Vijiaratnam N, Simuni T, Bandmann O, Morris HR, Foltynie T (2021). Progress towards therapies for disease modification in Parkinson’s disease. Lancet Neurol.

[21] Cummings J (2017). Disease modification and neuroprotection in neurodegenerative disorders. Transl Neurodegener.

[22] European Medicines Agency. Guideline on clinical investigation of medicinal products in the treatment of Parkinson’ s disease. EMA/CHMP/330418/2012. 2012;1–16.

[23] Food and Drug Administration. Early Alzheimer’s disease: developing drugs for treatment; Draft Guidance for Industry. 2018.

[24] Berg D, Postuma RB, Adler CH, Bloem BR, Chan P, Dubois B (2015). MDS research criteria for prodromal Parkinson’s disease. Mov Disord.

[25] Kim IY, O’Reilly ÉJ, Hughes KC, Gao X, Schwarzschild MA, Hannan MT (2018). Integration of risk factors for Parkinson disease in 2 large longitudinal cohorts. Neurology.

[26] Schrag A, Anastasiou Z, Ambler G, Noyce A, Walters K (2019). Predicting diagnosis of Parkinson’s disease: a risk algorithm based on primary care presentations. Mov Disord.

[27] Noyce AJ, R’Bibo L, Peress L, Bestwick JP, Adams-Carr KL, Mencacci NE (2017). PREDICT-PD: an online approach to prospectively identify risk indicators of Parkinson’s disease. Mov Disord.

[28] Noyce AJ, Bestwick JP, Silveira-Moriyama L, Hawkes CH, Knowles CH, Hardy J (2014). PREDICT-PD: identifying risk of Parkinson’s disease in the community: methods and baseline results. J Neurol Neurosurg Psychiatry.

[29] Heinzel S, Berg D, Gasser T, Chen H, Yao C, Postuma RB (2019). Update of the MDS research criteria for prodromal Parkinson’s disease. Mov Disord.

[30] Darweesh SKL, Koudstaal PJ, Stricker BH, Hofman A, Steyerberg EW, Ikram MA (2016). Predicting Parkinson disease in the community using a nonmotor risk score. Eur J Epidemiol.

[31] Marini K, Mahlknecht P, Tutzer F, Stockner H, Gasperi A, Djamshidian A (2020). Application of a simple Parkinson’s disease risk score in a longitudinal population-based cohort. Mov Disord.

[32] Bestwick JP, Auger SD, Simonet C, Rees RN, Rack D, Jitlal M (2021). Improving estimation of Parkinson’s disease risk-the enhanced PREDICT-PD algorithm. NPJ Park Dis.

[33] Pilotto A, Heinzel S, Suenkel U, Lerche S, Brockmann K, Roeben B (2017). Application of the movement disorder society prodromal Parkinson’s disease research criteria in 2 independent prospective cohorts. Mov Disord.

[34] Mahlknecht P, Gasperi A, Djamshidian A, Kiechl S, Stockner H, Willeit P (2018). Performance of the Movement Disorders Society criteria for prodromal Parkinson’s disease: a population-based 10-year study. Mov Disord.

[35] Giagkou Nikolaos, Maraki Maria I., Yannakoulia Mary, Kosmidis Mary H., Dardiotis Efthimios, Hadjigeorgiou Georgios M., Sakka Paraskevi, Ntanasi Eva, Anastasiou Costas A., Xiromerisiou Georgia, Stefanis Leonidas, Scarmeas Nikolaos, Stamelou Maria (2020). A Prospective Validation of the Updated Movement Disorders Society Research Criteria for Prodromal Parkinson's Disease. Movement Disorders.

[36] Marini Kathrin, Seppi Klaus, Tschiderer Lena, Kiechl Stefan, Stockner Heike, Willeit Peter, Willeit Johann, Djamshidian Atbin, Rungger Gregorio, Poewe Werner, Mahlknecht Philipp (2021). Application of the Updated Movement Disorder Society Criteria for Prodromal Parkinson's Disease to a Population‐Based 10‐Year Study. Movement Disorders.

[37] Fereshtehnejad S-M, Montplaisir JY, Pelletier A, Gagnon J-F, Berg D, Postuma RB (2017). Validation of the MDS research criteria for prodromal Parkinson’s disease: longitudinal assessment in a REM sleep behavior disorder (RBD) cohort. Mov Disord.

[38] Mirelman A, Saunders-Pullman R, Alcalay RN, Shustak S, Thaler A, Gurevich T (2018). Application of the movement disorder society prodromal criteria in healthy G2019S-LRRK2 carriers. Mov Disord.

[39] Siderowf A, Jennings D, Eberly S, Oakes D, Hawkins KA, Ascherio A (2012). Impaired olfaction and other prodromal features in the Parkinson At-Risk Syndrome study. Mov Disord.

[40] Jennings D, Siderowf A, Stern M, Seibyl J, Eberly S, Oakes D (2014). Imaging prodromal Parkinson disease: the Parkinson Associated Risk Syndrome Study. Neurology.

[41] Berg D, Godau J, Seppi K, Behnke S, Liepelt-Scarfone I, Lerche S (2013). The PRIPS study: screening battery for subjects at risk for Parkinson’s disease. Eur J Neurol.

[42] Berg D, Marek K, Ross GW, Poewe W (2012). Defining at-risk populations for Parkinson’s disease: lessons from ongoing studies. Mov Disord.

[43] Jennings D, Siderowf A, Stern M, Seibyl J, Eberly S, Oakes D (2017). Conversion to Parkinson disease in the PARS hyposmic and dopamine transporter-deficit prodromal cohort. JAMA Neurol.

[44] Postuma RB, Gagnon J-F, Bertrand J-A, Génier Marchand D, Montplaisir JY (2015). Parkinson risk in idiopathic REM sleep behavior disorder: preparing for neuroprotective trials. Neurology.

[45] Postuma RB, Pelletier A, Berg D, Gagnon JF, Escudier F, Montplaisir J (2016). Screening for prodromal Parkinson’s disease in the general community: a sleep-based approach. Sleep Med.

[46] Pujol M, Pujol J, Alonso T, Fuentes A, Pallerola M, Freixenet J, et al. Idiopathic REM sleep behavior disorder in the elderly Spanish community: a primary care center study with a two-stage design using video-polysomnography. Sleep Med. 2017;40:116–21. 10.1016/j.sleep.2017.07.021 29042180

[47] Bušková J, Ibarburu V, Šonka K, Růžička E (2016). Screening for REM sleep behavior disorder in the general population. Sleep Med.

[48] Mahlknecht P, Seppi K, Frauscher B, Kiechl S, Willeit J, Stockner H (2015). Probable RBD and association with neurodegenerative disease markers: a population-based study. Mov Disord.

[49] Toffoli M, Vieira SRL, Schapira AHV (2020). Genetic causes of PD: a pathway to disease modification. Neuropharmacology.

[50] von Linstow CU, Gan-Or Z, Brundin P (2020). Precision medicine in Parkinson’s disease patients with LRRK2 and GBA risk variants—let’s get even more personal. Transl Neurodegener.

[51] Heim B, Krismer F, De Marzi R, Seppi K (2017). Magnetic resonance imaging for the diagnosis of Parkinson’s disease. J Neural Transm.

[52] Saeed U, Compagnone J, Aviv RI, Strafella AP, Black SE, Lang AE (2017). Imaging biomarkers in Parkinson’s disease and Parkinsonian syndromes: current and emerging concepts. Transl Neurodegener.

[53] Mitchell T, Lehéricy S, Chiu SY, Strafella AP, Stoessl AJ, Vaillancourt DE (2021). Emerging neuroimaging biomarkers across disease stage in Parkinson disease: a review. JAMA Neurol.

[54] De Marzi R, Seppi K, Högl B, Müller C, Scherfler C, Stefani A (2016). Loss of dorsolateral nigral hyperintensity on 3.0 tesla susceptibility-weighted imaging in idiopathic rapid eye movement sleep behavior disorder. Ann Neurol.

[55] Mahlknecht P, Krismer F, Poewe W, Seppi K (2017). Meta-analysis of dorsolateral nigral hyperintensity on magnetic resonance imaging as a marker for Parkinson’s disease. Mov Disord.

[56] Barber TR, Griffanti L, Bradley KM, McGowan DR, Lo C, Mackay CE (2020). Nigrosome 1 imaging in REM sleep behavior disorder and its association with dopaminergic decline. Ann Clin Transl Neurol.

[57] Rossi M, Candelise N, Baiardi S, Capellari S, Giannini G, Orrù CD (2020). Ultrasensitive RT-QuIC assay with high sensitivity and specificity for Lewy body-associated synucleinopathies. Acta Neuropathol.

[58] Iranzo A, Fairfoul G, Ayudhaya ACN, Serradell M, Gelpi E, Vilaseca I (2021). Detection of α-synuclein in CSF by RT-QuIC in patients with isolated rapid-eye-movement sleep behaviour disorder: a longitudinal observational study. Lancet Neurol.

[59] Wang Z, Becker K, Donadio V, Siedlak S, Yuan J, Rezaee M (2020). Skin α-synuclein aggregation seeding activity as a novel biomarker for Parkinson disease. JAMA Neurol.

[60] Kuzkina A, Bargar C, Schmitt D, Rößle J, Wang W, Schubert A-L (2021). Diagnostic value of skin RT-QuIC in Parkinson’s disease: a two-laboratory study. NPJ Park Dis.

[61] Manne S, Kondru N, Jin H, Serrano GE, Anantharam V, Kanthasamy A (2020). Blinded RT-QuIC analysis of α-synuclein biomarker in skin tissue from Parkinson’s disease patients. Mov Disord.

[62] Stefani A, Iranzo A, Holzknecht E, Perra D, Bongianni M, Gaig C (2021). Alpha-synuclein seeds in olfactory mucosa of patients with isolated REM sleep behaviour disorder. Brain.

[63] Parkinson Study Group (1993). Effects of tocopherol and deprenyl on the progression of disability in early Parkinson’s disease. N Engl J Med.

[64] Fahn S, Oakes D, Shoulson I, Kieburtz K, Rudolph A, Lang A (2004). Levodopa and the progression of Parkinson’s disease. N Engl J Med.

[65] Verschuur CVM, Suwijn SR, Boel JA, Post B, Bloem BR, van Hilten JJ (2019). Randomized delayed-start trial of Levodopa in Parkinson’s disease. N Engl J Med.

[66] Schapira AHV, McDermott MP, Barone P, Comella CL, Albrecht S, Hsu HH (2013). Pramipexole in patients with early Parkinson’s disease (PROUD): a randomised delayed-start trial. Lancet Neurol.

[67] Olanow CW, Rascol O, Hauser R, Feigin PD, Jankovic J, Lang A (2009). A double-blind, delayed-start trial of rasagiline in Parkinson’s disease. N Engl J Med.

[68] Writing Group for the NINDS Exploratory Trials in Parkinson Disease (NET-PD) Investigators, Kieburtz K, Tilley BC, Elm JJ, Babcock D, Hauser R, et al. Effect of creatine monohydrate on clinical progression in patients with Parkinson disease: a randomized clinical trial. JAMA. 2015;313:584–93. 10.1001/jama.2015.120 PMC4349346 25668262

[69] Athauda D, Maclagan K, Skene SS, Bajwa-Joseph M, Letchford D, Chowdhury K (2017). Exenatide once weekly versus placebo in Parkinson’s disease: a randomised, double-blind, placebo-controlled trial. Lancet.

[70] Thibault L, Rascol O, Corvol JC, Ferreira J, Defebvre L, Deplanque D (2017). New perspectives on study designs for evaluating neuroprotection in Parkinson’s disease. Mov Disord.

[71] Kieburtz K (2016). Treating neurodegenerative disease before illness: a challenge for the 21st century. Lancet Neurol.

[72] Eberling JL, Dave KD, Frasier M (2013). α-synuclein imaging: a critical need for Parkinson’s disease research. J Parkinsons Dis.

[73] Iranzo A, Santamaría J, Valldeoriola F, Serradell M, Salamero M, Gaig C (2017). Dopamine transporter imaging deficit predicts early transition to synucleinopathy in idiopathic rapid eye movement sleep behavior disorder. Ann Neurol.

[74] Darweesh SKL, Verlinden VJA, Stricker BH, Hofman A, Koudstaal PJ, Ikram MA (2017). Trajectories of prediagnostic functioning in Parkinson’s disease. Brain.

[75] Postuma RB, Lang AE, Gagnon JF, Pelletier A, Montplaisir JY (2012). How does parkinsonism start? Prodromal parkinsonism motor changes in idiopathic REM sleep behaviour disorder. Brain.

[76] Alotaibi F, Pelletier A, Gagnon J-F, Montplaisir JY, Postuma RB (2019). Prodromal marker progression in idiopathic rapid eye movement sleep behavior disorder: sample size for clinical trials. Mov Disord.

[77] Latourelle JC, Beste MT, Hadzi TC, Miller RE, Oppenheim JN, Valko MP (2017). Large-scale identification of clinical and genetic predictors of motor progression in patients with newly diagnosed Parkinson’s disease: a longitudinal cohort study and validation. Lancet Neurol.

[78] Holden SK, Finseth T, Sillau SH, Berman BD (2018). Progression of MDS-UPDRS scores over five years in de novo Parkinson disease from the Parkinson’s Progression Markers Initiative Cohort. Mov Disord Clin Pract.

[79] Simuni T, Siderowf A, Lasch S, Coffey CS, Caspell-Garcia C, Jennings D (2018). Longitudinal change of clinical and biological measures in early Parkinson’s disease: Parkinson’s progression markers initiative cohort. Mov Disord.

[80] Simuni T, Caspell-Garcia C, Coffey CS, Weintraub D, Mollenhauer B, Lasch S (2018). Baseline prevalence and longitudinal evolution of non-motor symptoms in early Parkinson’s disease: the PPMI cohort. J Neurol Neurosurg Psychiatry.

[81] Liepelt-Scarfone I, Brändle B, Yilmaz R, Gauss K, Schaeffer E, Timmers M (2017). Progression of prodromal motor and non-motor symptoms in the premotor phase study—2-year follow-up data. Eur J Neurol.

[82] Iranzo A, Valldeoriola F, Lomeña F, Molinuevo JL, Serradell M, Salamero M (2011). Serial dopamine transporter imaging of nigrostriatal function in patients with idiopathic rapid-eye-movement sleep behaviour disorder: a prospective study. Lancet Neurol.

[83] Burciu RG, Ofori E, Archer DB, Wu SS, Pasternak O, McFarland NR (2017). Progression marker of Parkinson’s disease: a 4-year multi-site imaging study. Brain.

[84] Biondetti E, Santin MD, Valabrègue R, Mangone G, Gaurav R, Pyatigorskaya N, et al. The spatiotemporal changes in dopamine, neuromelanin and iron characterizing Parkinson’s disease. Brain. 2021;2–35. 10.1093/brain/awab191 PMC8634084 33978742

[85] Matsuura K, Maeda M, Tabei K-I, Umino M, Kajikawa H, Satoh M (2016). A longitudinal study of neuromelanin-sensitive magnetic resonance imaging in Parkinson’s disease. Neurosci Lett.

[86] Mollenhauer B, Caspell-Garcia CJ, Coffey CS, Taylor P, Singleton A, Shaw LM (2019). Longitudinal analyses of cerebrospinal fluid α-synuclein in prodromal and early Parkinson’s disease. Mov Disord.

[87] Mollenhauer B, Caspell-Garcia CJ, Coffey CS, Taylor P, Shaw LM, Trojanowski JQ (2017). Longitudinal CSF biomarkers in patients with early Parkinson disease and healthy controls. Neurology.

[88] Poewe W, Mahlknecht P (2009). The clinical progression of Parkinson’s disease. Parkinsonism Relat Disord.

[89] Poewe W, Mahlknecht P (2020). Pharmacologic treatment of motor symptoms associated with Parkinson disease. Neurol Clin.

[90] Peterschmitt MJ, Hidemoto S, Taku H, Thomas G, Isaacson SH, Gaemers SJM, Minini P (2021). Safety, pharmacokinetics, and pharmacodynamics of oral venglustat in patients with Parkinson's disease and a GBA mutation: results from part 1 of the randomized, double-blinded, placebo-controlled MOVES-PD trial. J Parkinsons Dis.

[91] Taylor K, Lipsmeier F, Volkova-Volkmar E, Rukina D, Anzures Cabrera J, Essioux L (2021). Prasinezumab reduced progression of Parkinson’s disease motor features measured by Roche PD Mobile Application v2 sensor features: PASADENA Phase II Part 1. Mov Disord.

[92] Hutchison RM, Evans KC, Fox T, Yang M, Barakos J, Bedell BJ (2021). Evaluating dopamine transporter imaging as an enrichment biomarker in a phase 2 Parkinson's disease trial. BMC Neurol.

[93] Alexander GC, Knopman DS, Emerson SS, Ovbiagele B, Kryscio RJ, Perlmutter JS (2021). Revisiting FDA approval of Aducanumab. N Engl J Med.

[94] Berg D, Borghammer P, Fereshtehnejad SM, Heinzel S, Horsager J, Schaeffer E (2021). Prodromal Parkinson disease subtypes - key to understanding heterogeneity. Nat Rev Neurol.

[95] Fereshtehnejad S-M, Zeighami Y, Dagher A, Postuma RB (2017). Clinical criteria for subtyping Parkinson’s disease: biomarkers and longitudinal progression. Brain.

[96] De Pablo-Fernández E, Lees AJ, Holton JL, Warner TT (2019). Prognosis and neuropathologic correlation of clinical subtypes of Parkinson disease. JAMA Neurol.

[97] Borghammer P, Van Den Berge N (2019). Brain-first versus gut-first Parkinson’s disease: a hypothesis. J Parkinsons Dis.

[98] Horsager J, Andersen KB, Knudsen K, Skjærbæk C, Fedorova TD, Okkels N (2020). Brain-first versus body-first Parkinson’s disease: a multimodal imaging case-control study. Brain.

[99] Knudsen K, Fedorova TD, Hansen AK, Sommerauer M, Otto M, Svendsen KB (2018). In-vivo staging of pathology in REM sleep behaviour disorder: a multimodality imaging case-control study. Lancet Neurol.

[100] Ranson JM, Rittman T, Hayat S, Brayne C, Jessen F, Blennow K (2021). Modifiable risk factors for dementia and dementia risk profiling. A user manual for Brain Health Services-part 2 of 6. Alzheimers Res Ther..

[101] Marras C, Canning CG, Goldman SM (2019). Environment, lifestyle, and Parkinson’s disease: implications for prevention in the next decade. Mov Disord.

[102] Maraki MI, Yannakoulia M, Stamelou M, Stefanis L, Xiromerisiou G, Kosmidis MH (2019). Mediterranean diet adherence is related to reduced probability of prodromal Parkinson’s disease. Mov Disord.

[103] Schaeffer E, Rogge A, Nieding K, Helmker V, Letsch C, Hauptmann B (2020). Patients’ views on the ethical challenges of early Parkinson disease detection. Neurology.

[104] Wilson JM, Jungner YG (1968). Principles and practice of mass screening for disease. Bol Oficina Sanit Panam.

[105] Andermann A, Blancquaert I, Beauchamp S, Déry V (2008). Revisiting Wilson and Jungner in the genomic age: a review of screening criteria over the past 40 years. Bull World Health Organ.

[106] Verweij M (1999). Medicalization as a moral problem for preventive medicine. Bioethics.

[107] van Dam L, Bretthauer M (2014). Ethical issues in colorectal cancer screening. Best Pract Res Clin Gastroenterol.

[108] Jørgensen KJ, Gøtzsche PC (2009). Overdiagnosis in publicly organised mammography screening programmes: systematic review of incidence trends. BMJ.

[109] Kramer BS, Croswell JM (2009). Cancer screening: the clash of science and intuition. Annu Rev Med.

[110] Eaden J, Mayberry MK, Sherr A, Mayberry JF (2001). Screening: the legal view. Public Health.

[111] Schaeffer E, Toedt I, Köhler S, Rogge A, Berg D (2021). Risk disclosure in prodromal Parkinson’s disease. Mov Disord.

